# AGER-Mediated Lipid Peroxidation Drives Caspase-11 Inflammasome Activation in Sepsis

**DOI:** 10.3389/fimmu.2019.01904

**Published:** 2019-08-08

**Authors:** Ruochan Chen, Shan Zhu, Ling Zeng, Qingde Wang, Yi Sheng, Borong Zhou, Daolin Tang, Rui Kang

**Affiliations:** ^1^The Third Affiliated Hospital, Guangzhou Medical University, Guangzhou, China; ^2^Department of Infectious Diseases and State Key Lab of Viral Hepatitis, Xiangya Hospital, Central South University, Changsha, China; ^3^Department of Pediatrics, The Third Xiangya Hospital, Central South University, Changsha, China; ^4^State Key Laboratory of Trauma, Burns and Combined Injury, Research Institute of Surgery, Research Institute for Traffic Medicine of People's Liberation Army, Daping Hospital, Third Military Medical University, Chongqing, China; ^5^Department of Surgery, University of Pittsburgh, Pittsburgh, PA, United States; ^6^Department of Obstetrics, Gynecology and Reproductive Sciences, University of Pittsburgh, Pittsburgh, PA, United States; ^7^Department of Surgery, UT Southwestern Medical Center, Dallas, TX, United States

**Keywords:** DAMP, AGER, ALOX5, caspase-11, sepsis, inflammasome, lipid peroxidation, LPS

## Abstract

Inflammasome activation can trigger an inflammatory and innate immune response through the release of cytokines and induction of pyroptosis. A dysfunctional inflammasome has been implicated in the development of human pathologies, including sepsis and septic shock. Here, we show that advanced glycosylation end-product specific receptor (AGER/RAGE) is required for caspase-11 inflammasome activation in macrophages. A nuclear damage-associated molecular pattern (nDAMP) complex, including high-mobility group box 1, histone, and DNA, can promote caspase-11-mediated gasdermin D cleavage, interleukin 1β proteolytic maturation, and lactate dehydrogenase release. The inhibition of AGER-mediated lipid peroxidation via arachidonate 5-lipoxygenase (ALOX5) limits caspase-11 inflammasome activation and pyroptosis in macrophages in response to nDAMPs or cytosolic lipopolysaccharide. Importantly, the pharmacologic inhibition of the AGER-ALOX5 pathway or global depletion (*Ager*^−/−^) or conditional depletion of *AGER* in myeloid cells (*Ager*^*Mye*−/−^) protects against lipopolysaccharide-induced septic death in poly(I:C)-primed mice. These data identify a molecular basis for caspase-11 inflammasome activation and provide a potential strategy to treat sepsis.

## Introduction

Inflammation is an immune system response to danger signals, including foreign pathogen-associated molecular patterns (PAMPs) and endogenous damage-associated molecular patterns (DAMPs). These danger signals are recognized by specific pattern recognition receptors to trigger various immune responses as well as host cell death. Extracellular lipopolysaccharide (LPS), as a typical PAMP from gram-negative bacteria, can bind toll-like receptor 4 (TLR4) on the cell surface to induce cytokine or chemokine expression (1). In contrast, intracellular LPS is able to cause caspase-11-dependent and caspase-1-independent inflammasome activation (2–5). The activation of caspase-11 by cytosolic LPS drives the production of mature interleukin (IL)-1 family cytokines (e.g., IL-1β and IL-18), as well as gasdermin D (GSDMD) cleavage, which is responsible for the induction of pyroptosis, a form of regulated cell death mainly in macrophages and monocytes (6–9). The caspase-11-dependent inflammasome is deregulated in the context of various human pathologies, including infection and tissue injury. *Casp11*- or *Gsdmd*-deficient mice are protected from lethal endotoxemia or polymicrobial-induced septic shock (2, 4, 6, 10). Thus, caspase-11 inflammasome and its modulation have considerable potential as a therapeutic approach in lethal inflammation (11).

Nuclear DAMPs (nDAMPs), such as high mobility group box 1 (HMGB1), histone, and DNA are components or regulators of chromosome in eukaryotes. The release of nDAMPs play a pathologic role in the linking of genomic instability, DNA damage, and the inflammation response in disease (12, 13). In addition to exerting a singular effect, these nDAMPs usually are found as a complex in serum to mediate the immune response in certain human diseases such as systemic lupus erythematosus (14). Our previous study showed that HMGB1-histone-DNA complex (HHD) causes regulated cell death in macrophages (15). However, its role in inflammasome activation remains unclear. In the present study, we further demonstrated that caspase-11-mediated GSDMD cleavage is required for HHD-induced pyroptosis in macrophages. This process requires advanced glycosylation end-product specific receptor (AGER/RAGE)-mediated lipid peroxidation. Importantly, we demonstrate that the global or conditional deletion of *Ager* in myeloid cells protects against caspase-11-associated septic death in mice. Thus, targeting the AGER pathway could be a promising strategy for the prevention and treatment of inflammasome-associated disease.

## Materials and Methods

### Antibodies and Reagents

The antibodies to caspase-11 (#14340) and actin (#3700) were obtained from Cell Signaling Technology (Danvers, MA, USA). The antibody to IL-1β (#AF-401-NA) was obtained from R&D Systems (Minneapolis, MN, USA). The antibodies to GSDMD (#Sc-393656) and 5-lipoxygenase (ALOX5; #sc-515821) were obtained from Santa Cruz Biotechnology (Dallas, Texas, USA). Recombinant mouse HMGB1 protein (#764004) was obtained from BioLegend (San Diego, CA, USA). Mouse genomic DNA (#N4004) was obtained from New England BioLabs (Ipswich, MA, USA). A mixture of histones H1, H2A, H2B, H3, and H4 were isolated from calf thymus (#10223565001) and obtained from Sigma-Aldrich (St. Louis, MO, USA). LPS (*Escherichia coli* LPS 0111:B4; #L4391) was obtained from Sigma-Aldrich. FPS-ZM1(#553030) was obtained from EMD Millipore (Billerica, MA, USA). Zileuton (#S1443) was obtained from Selleck Chemicals (Houston, TX, USA). Poly(I:C) (#31852-29-6) was obtained from InvivoGen (San Diego, CA, USA).

### Cell Culture and Treatment

Immortalized wild-type (WT) and *Nlrp3*^−/−^ bone-marrow-derived macrophages (BMDMs) were a kind gift from Dr. Kate Fitzgerald. BMDMs from *Casp11*^−/−^ mice were obtained using 30% L929-cell conditioned medium as a source of granulocyte/macrophage colony stimulating factor (16). CRISPR/Cas9-mediated *Gsdmd*^−/−^ BMDMs were a kind gift from Dr. Derek Abbott. These cells were cultured in Dulbecco's Modified Eagle's Medium (DMEM; #11995073, Thermo Fisher Scientific, Waltham, MA, USA) supplemented with 10% heat-inactivated fetal bovine serum (#TMS-013-B, EMD Millipore) and 1% penicillin and streptomycin (#15070-063, Thermo Fisher Scientific) at 37°C, 95% humidity, and 5% CO_2_. Cells were primed with LPS (200 ng/ml, 6 h) and then stimulated by HHD [HMGB1 (500 ng/mL) + histone (500 ng/mL) + genomic DNA (500 ng/mL), 16 h], LPS electroporation (1 μg, 16 h), or *E. coli* [multiplicity of infection (MOI) = 25, 16 h] infection. All cells used were authenticated using short tandem repeat profiling and mycoplasma testing was negative.

### LPS Transfection

To stimulate caspase-11 non-canonical inflammasome activation, LPS was electroporated into indicated cells using the Neon Transfection System (Thermo Fisher Scientific) according to the manufacturer's protocol. Briefly, BMDMs were electroporated with LPS in buffer R (#MPK10025, Thermo Fisher Scientific) under pulse voltage 1,400 V, pulse width 10 ms, and pulse number 2.

### Bacterial Infection

*E. coli* (#11775) were obtained from American Type Culture Collection (Manassas, VA, USA) and then added to cells at an MOI of 25 in media without antibiotics. After 30 min, cells were washed and incubated for 1.5 h at 37°C in fresh medium supplemented with gentamicin (100 μg/ml, #G1397, Sigma-Aldrich) to kill extracellular bacteria.

### Mouse Model

*Ager*^−/−^ mice (C57BL/6) were a gift from Dr. Angelica Bierhaus. *Ager*^−/−*Mye*^ mice were generated by crossing *Ager*^*flox*/*flox*^ mice with *LysM-Cre* mice (#004781, The Jackson Laboratory, Bar Harbor, ME, USA). The *Ager*^*flox*/*flox*^ mice were created by inserting *loxP* sites within intron 1 and intron 2, flanking exon 11 of Ager. Septic shock was induced in male or female C57BL/6 mice (8 to 10 weeks old, 22 to 26 g body weight). These mice were primed with poly(I:C) (10 mg/kg, i.p.) and then challenged 6 h later with LPS (2 mg/kg, i.p.) (2). Animal studies were approved by our institutional animal care and use committees and conducted in accordance with Association for Assessment and Accreditation of Laboratory Animal Care guidelines (http://www.aaalac.org/). Mice were housed in individually ventilated cages and were maintained in specific pathogen-free facilities.

### Cytotoxicity Assay

Lactate dehydrogenase (LDH) release was evaluated using an LDH Assay Kit (#ab102526) from Abcam (Cambridge, MA, USA) according to the manufacturer's instructions. The released LDH was normalized to total LDH content measured in 1% Triton X-100–permeabilized samples of indicated cells.

### Lipid Peroxidation Assay

The relative malondialdehyde (MDA) concentration in cells was assessed using a Lipid Peroxidation (MDA) Assay Kit (#ab118970, Abcam) according the manufacturer's instructions. Briefly, MDA in the sample reacts with thiobarbituric acid (TBA) to generate a MDA-TBA adduct. The MDA-TBA adduct can be easily quantified colorimetrically (OD = 532 nm) or fluorometrically (Ex/Em = 532/553 nm). The concentration of 4-hydroxynonenal (4-HNE) was assessed using an ELISA Kit (#LS-F28410-1) from LifeSpan BioSciences (Seattle, WA, USA) according to the manufacturer's instructions.

### Biochemical Assay

Measurements of serum tissue enzymes (creatine kinase [CK], blood urea nitrogen [BUN], and alanine aminotransferase [ALT]) were performed using an IDEXX Catalyst Dx Chemistry Analyzer (IDEXX, Westbrook, ME, USA) (17).

### Cytokine Analysis

Commercially available enzyme-linked immunosorbent assay (ELISA) kits were used to measure the concentrations of IL-1β (#MLB00C, R&D Systems), IL-18 (#7625, R&D Systems), IL-1α (#MLA00, R&D Systems), tumor necrosis factor (TNF; #MTA00B, R&D Systems), IL-6 (#M6000B, R&D Systems), and IL-12 p70 (#M1270, R&D Systems) in cell culture medium or serum according to the manufacturer's instructions.

### Lipoxygenase Activity Assay

A commercially available Lipoxygenase Activity Assay Kit (#K978-100) from BioVision (Milpitas, CA, USA) was used to measure lipoxygenase activity in cell lysate according to the manufacturer's instructions. Lipoxygenase converts the substrate to an intermediate that reacts with the probe generating a fluorescent product. The increase in fluorescent signal can be recorded at Ex/Em 500/536 nm and is directly proportional to lipoxygenase activity.

### RNAi and Plasmid Transfection

ON-TARGETplus SMART pool small interfering RNAs (siRNAs) against mouse *Alox5* gene (#L-065695-01-0005) was purchased from Dharmacon (Lafayette, CO, USA). This pool was a mixture of four siRNAs provided as a single reagent. GSDMD cleavage mutant (D275A) and full-length WT GSDMD plasmids were a kind gift from Dr. Feng Shao. The Neon Electroporation System (Thermo Fisher Scientific) was used to deliver siRNAs or plasmid DNA into BMDMs. Transfected cells were recovered in complete DMEM. The medium was replaced at 3 h post-electroporation. The cells were cultured for 48 h before further examination.

### Western Blot

Western blot was used to analyze protein expression as described previously (18). In brief, after extraction, proteins in cell lysates were first resolved by 4–12% Criterion XT Bis-Tris gel electrophoresis (#3450124, Bio-Rad Laboratories, Hercules, CA, USA) and then transferred to polyvinylidene difluoride membranes. After blocking with 5% non-fat dry milk or bovine serum albumin, the membranes were subsequently incubated with the primary antibody (1:100–1:1000). After incubation with peroxidase-conjugated secondary antibodies (1:1000–1:2000), the signals were visualized using enhanced chemiluminescence (#32106, Thermo Fisher Scientific).

### Histologic Examination

After fixation in 4% phosphate buffered formaldehyde, tissues were embedded in optimum cutting temperature cryomedium (Sakura Finetek, Torrance, CA, USA) and cut into 4 μm sections. Hematoxylin (modified Harris hematoxylin; Thermo Scientific) and eosin (eosin-Y; Thermo Scientific) staining was performed for each section to examine histomorphologic features (19).

### Statistical Analysis

Data are expressed as means ± SD. Unpaired Student's *t*-tests were used to compare the means of two groups. One-way analysis of variance (ANOVA) was used for comparison among the different groups. When an ANOVA was significant, *post hoc* testing of differences between groups was performed using the least significant difference (LSD) test. The Kaplan-Meier method was used to compare differences in mortality rates between groups. A *P* < 0.05 was considered statistically significant.

## Results

### Caspase-11 Is Required for Nuclear DAMP Complex-Induced Pyroptosis

**Figure 1:**
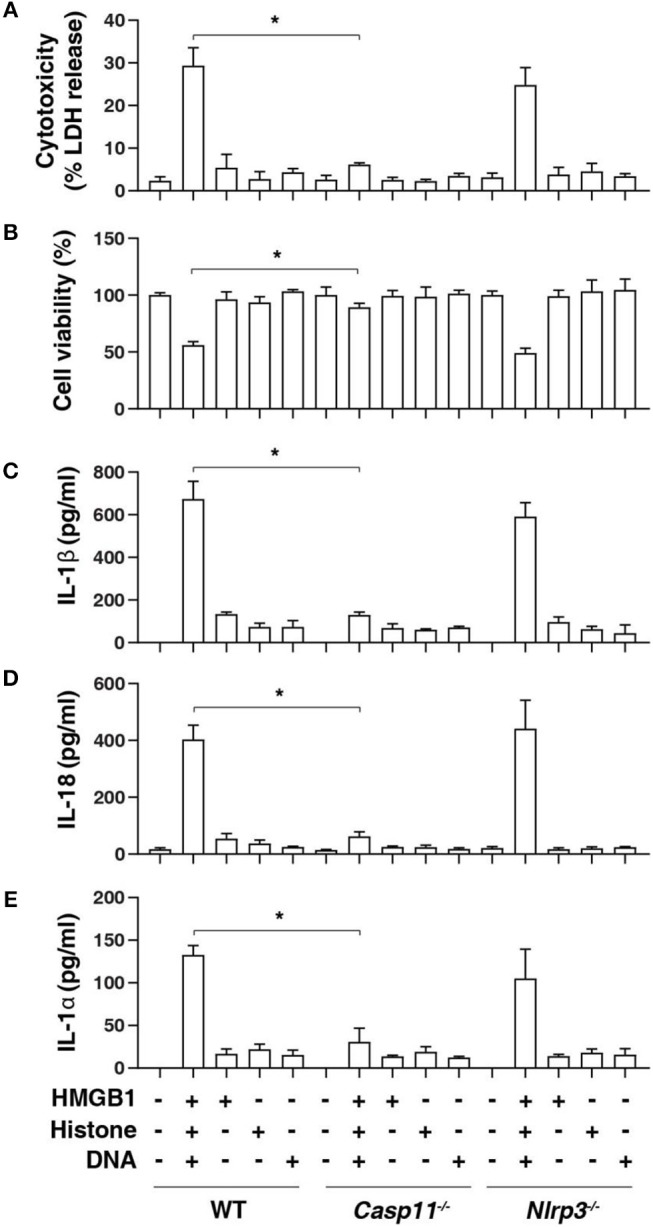
Caspase-11 is required for nuclear DAMP complex-induced pyroptosis. Indicated LPS-primed BMDMs were treated with HMGB1 (500 ng/mL), histone (500 ng/mL), and genomic DNA (500 ng/mL) for 16 h, then cytotoxicity **(A)**, cell viability **(B)**, IL-1β release **(C)**, IL-18 release **(D)**, and IL-1α release **(E)** were assayed. *n* = 3, data expressed as means ± SD of three independent experiments, *P < 0.05, *t* test.

Inflammasome activation in macrophages such as mouse BMDMs requires two steps. A first priming step contributes to the induction of the expression of inflammasome components, whereas a secondary sensing step facilitates the assembly and activation of an inflammasome (20). To determine the role of HHD in inflammasome activation, we treated LPS-primed BMDMs with HHD [HMGB1 (500 ng/mL) + histone (500 ng/mL) + genomic DNA (500 ng/mL)]. Following exposure to HHD for 16 h, cytotoxicity was determined with an LDH leakage assay. HHD (but not single stimuli) caused cytotoxicity (Figure 1A), cell viability inhibition (Figure 1B), IL-1β release (Figure 1C), IL-18 release (Figure 1D), and IL-1α release (Figure 1E) in LPS-primed BMDMs, indicating that HHD plays a potential role in inflammasome activation. Importantly, the deletion of *Casp11* [but not NLR family pyrin domain containing 3 (*Nlrp3*)] blocked HHD-induced cytotoxicity (Figure 1A), cell viability inhibition (Figure 1B), IL-1β release (Figure 1C), IL-18 release (Figure 1D), and IL-1α release (Figure 1E) in BMDMs. These findings indicate that caspase-11-dependent non classical inflammasome, but not caspase-1-dependent NLRP3 inflammasome, is an essential mediator of nDAMP-induced pyroptosis.

### GSDMD Is Required for Nuclear DAMP Complex-Induced Pyroptosis

**Figure 2:**
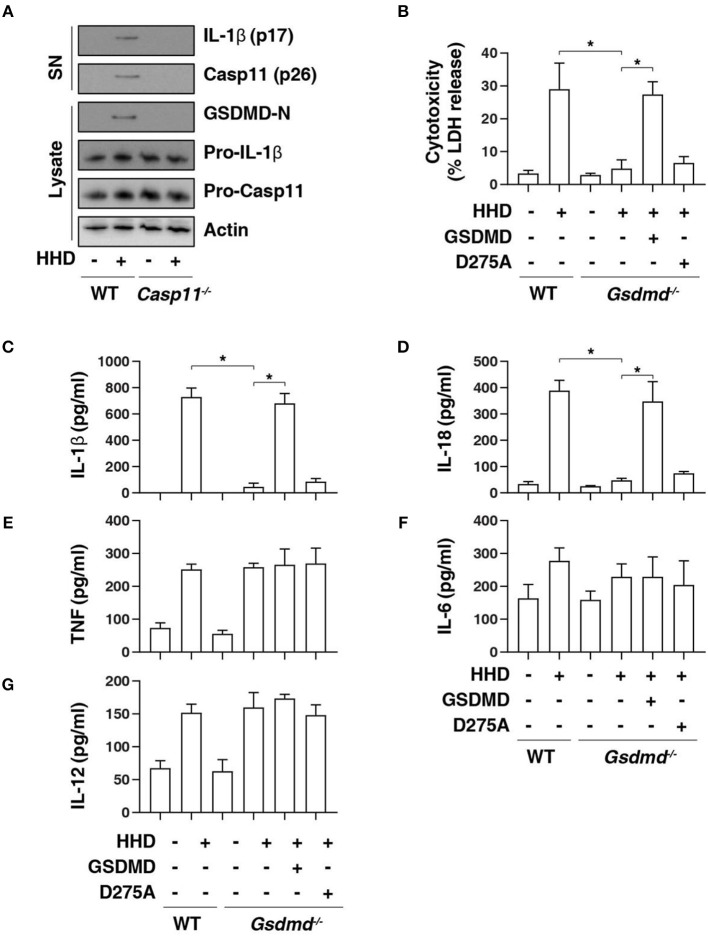
GSDMD is required for nuclear DAMP complex-induced pyroptosis. **(A)** Western blot analysis of indicated proteins in the supernatant (SN) or cell lysate in indicated LPS-primed BMDMs after HHD treatment (500 ng/mL, 16 h). **(B–G)** Analysis of cytotoxicity **(B)**, IL-1β **(C)**, IL-18 **(D)**, TNF **(E)**, IL-6 **(F)**, and IL-12 **(G)** release in indicated LPS-primed BMDMs after HHD treatment (500 ng/mL, 16 h) in the absence or presence of the overexpression of GSDMD WT or D275A cDNA. *n* = 3, data expressed as means ± SD of three independent experiments, *P < 0.05, *t* test. Western blot data represent two independent experiments.

GSDMD is a member of the gasdermin family and has been suggested to act as an effector of pyroptosis due to its role in the formation of membrane pores (8, 9, 21). Given that cleavage of GSDMD by inflammatory caspases determines pyroptotic cell death (6, 7, 22), we next determined whether HHD can cause GSDMD cleavage. Western blot analysis showed that HHD-induced GSDMD-N formation, proteolytic IL-1β maturation (p17), and caspase-11 (p26) activation was inhibited in LPS-primed *Casp11*^−/−^BMDMs (Figure 2A). Consequently, the deletion of *Gsdmd* (*Gsdmd*^−/−^) inhibited HHD-induced cytotoxicity in LPS-primed BMDMs (Figure 2B). A previous study demonstrated that GSDMD D275A is resistant to cleavage by caspase-11 and was unable to mediate LPS-induced pyroptosis (6, 7). Moreover, transfection with GSDMD cDNA, but not GSDMD-N cleavage mutant (D275A), restored HHD-induced cytotoxicity (Figure 2B), IL-1β release (Figure 2C), and IL-18 release (Figure 2D) to LPS-primed *Gsdmd*^−/−^ BMDMs. In contrast, GSDMD and D275A failed to affect HDD-induced TNF (Figure 2E), IL-6 (Figure 2F), and IL-12 (Figure 2G) release. Collectively, these findings indicate that GSDMD cleavage at D275 is required for HHD-induced pyroptosis.

### AGER Is Required for Caspase-11 Inflammasome Activation

**Figure 3:**
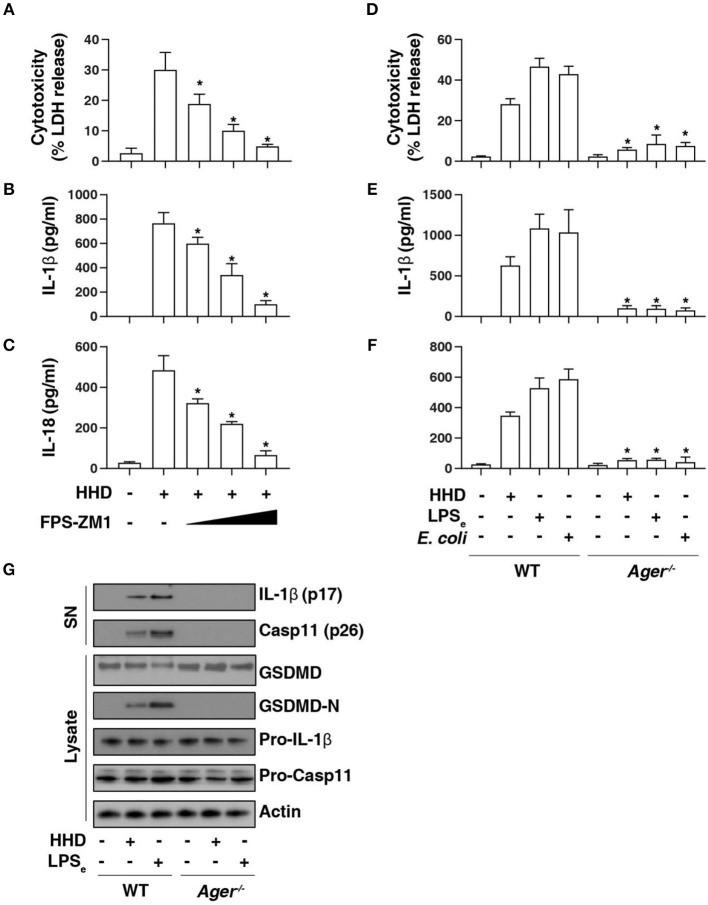
AGER is required for caspase-11 inflammasome activation. **(A–C)** Analysis of cytotoxicity **(A)**, IL-1β release **(B)**, and IL-18 release **(C)** in LPS-primed BMDMs after HHD treatment (500 ng/ml, 16 h) in the absence or presence of FPS-ZM1 (100 nM, 500 nM, and 1 μM). *n* = 3, data expressed as means ± SD of three independent experiments, *P < 0.05 vs. HHD group, *t* test. **(D–F)** Analysis of cytotoxicity **(D)**, IL-1β release **(E)**, and IL-18 release **(F)** in indicated LPS-primed BMDMs after HHD treatment (500 ng/ml, 16 h), LPS electroporation (LPS_e_; 1 μg, 16 h), or *E. coli* infection (MOI = 25, 16 h). *n* = 3, data expressed as means ± SD of three independent experiments, *P < 0.05 vs. WT group, *t* test. **(G)** Western blot analysis of indicated proteins in the supernatant (SN) or cell lysate in LPS-primed BMDMs after HHD treatment (500 ng/ml, 16 h) or LPS electroporation (LPS_e_; 1 μg, 16 h). Western blot data represent two independent experiments.

AGER is a multiple ligand receptor of nDAMPs, including HMGB1 (12), histone (13), and DNA (23). To address the role of AGER in caspase-11 inflammasome activation, we first used FPS-ZM1, a high-affinity AGER-specific inhibitor identified from high-throughput screenings in an experimental model of Alzheimer's disease (24). FPS-ZM1 dose-dependently inhibited HHD-induced cytotoxicity (Figure 3A), IL-1β release (Figure 3B), and IL-18 release (Figure 3C) in LPS-primed BMDMs. Consistent with AGER inhibitor, the deletion of *Ager* (*Ager*^−/−^) also blocked HHD-induced cytotoxicity (Figure 3D), IL-1β release (Figure 3E), and IL-18 release (Figure 3F) in LPS-primed BMDMs. Moreover, *Ager*^−/−^ BMDM was also resistant to LPS electroporation or *E. coli* infection-induced cytotoxicity (Figure 3D), IL-1β release (Figure 3E), and IL-18 release (Figure 3F) in LPS-primed BMDMs. These findings, combined with western blot analysis of GSDMD-N formation, proteolytic IL-1β maturation (p17), and caspase-11 (p26) activation (Figure 3G), indicate that AGER is a positive regulator of caspase-11 inflammasome activation and pyroptosis.

### AGER-Mediated Lipid Peroxidation Promotes Caspase-11 Inflammasome Activation

**Figure 4:**
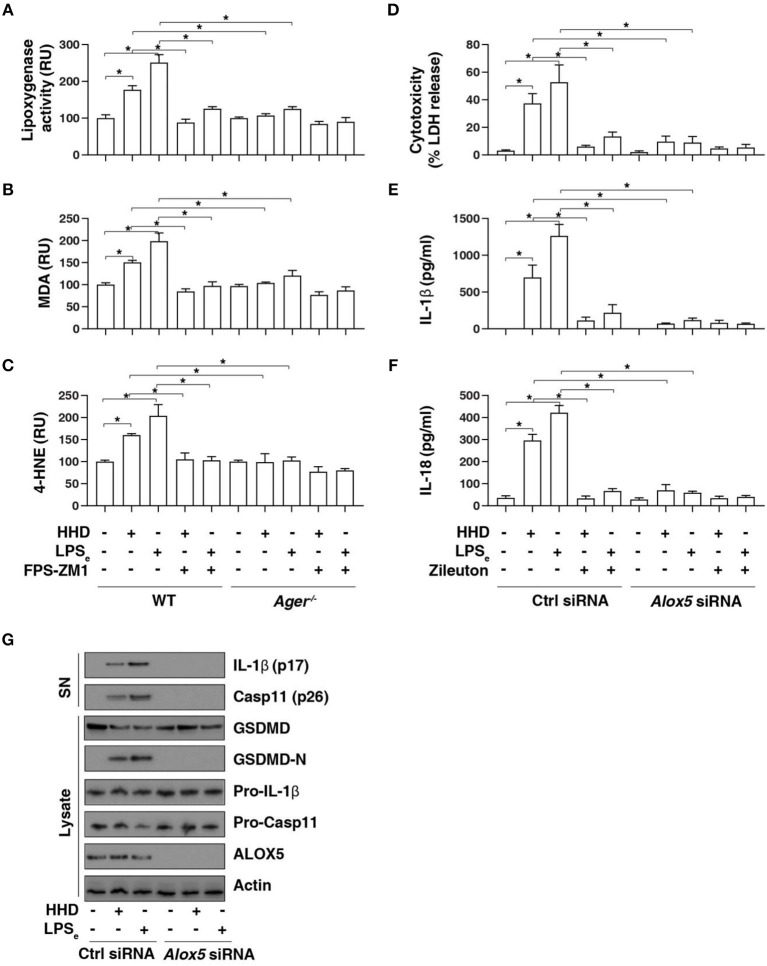
AGER-mediated lipid peroxidation promotes caspase-11 inflammasome activation. **(A–C)** Analysis of lipoxygenase activity **(A)**, MDA level **(B)**, and 4-HNE level **(C)** in indicated LPS-primed BMDMs after HHD treatment (500 ng/ml, 16 h) or LPS electroporation (LPS_e_; 1 μg, 16 h) in the absence or presence of FPS-ZM1 (1 μM). *n* = 3, data expressed as means ± SD of three independent experiments, *P < 0.05, *t* test. **(D–F)** Analysis of cytotoxicity **(D)**, IL-1β release **(E)**, and IL-18 release **(F)** in indicated LPS-primed BMDMs after HHD treatment (500 ng/ml, 16 h) or LPS electroporation (LPS_e_; 1 μg, 16 h) in the absence or presence of zileuton (5 μM). *n* = 3, data expressed as means ± SD of three independent experiments, *P < 0.05, *t* test. **(G)** Western blot analysis of indicated proteins in the supernatant (SN) or cell lysate in LPS-primed BMDMs after HHD treatment (500 ng/ml, 16 h) or LPS electroporation (LPS_e_; 1 μg, 16 h). Western blot data represent two independent experiments.

To further assess the role of AGER in pyroptosis, we examined lipid peroxidation, the process of oxidative degradation of lipids by lipoxygenase. The activity of lipoxygenase (Figure 4A) and level of the final products of lipid peroxidation, such as MDA (Figure 4B) and 4-HNE (Figure 4C) were increased in LPS-primed BMDMs following HHD treatment or LPS electroporation. In contrast, the pharmacological or genetic inhibition of AGER blocked HHD- or LPS electroporation-induced lipoxygenase activity (Figure 4A), MDA (Figure 4B) and 4-HNE (Figure 4C) production, indicating that AGER promotes lipid peroxidation in caspase-11 inflammasome activation.

To determine whether lipoxygenase is require for caspase-11 inflammasome activation, we treated cells with zileuton, an inhibitor of ALOX5 (25). Indeed, zileuton blocked HHD- or LPS electroporation-induced cytotoxicity (Figure 4D), IL-1β release (Figure 4E), and IL-18 release (Figure 4F) in LPS-primed BMDMs. Furthermore, knockdown of *Alox5* by siRNA-pool also blocked HHD- or LPS electroporation-induced cytotoxicity (Figure 4D), IL-1β release (Figure 4E), and IL-18 release (Figure 4F) in LPS-primed BMDMs. Western blot analysis further showed that GSDMD-N formation, proteolytic IL-1β maturation (p17), and caspase-11 (p26) activation was inhibited in LPS-primed *Alox5-*knockdown BMDMs in response to HHD or LPS electroporation (Figure 4G). Collectively, these findings indicate that AGER-mediated lipid peroxidation via ALOX5 promotes caspase-11 inflammasome activation.

### Targeting the AGER-ALOX5 Pathway Protects Against Septic Shock

**Figure 5:**
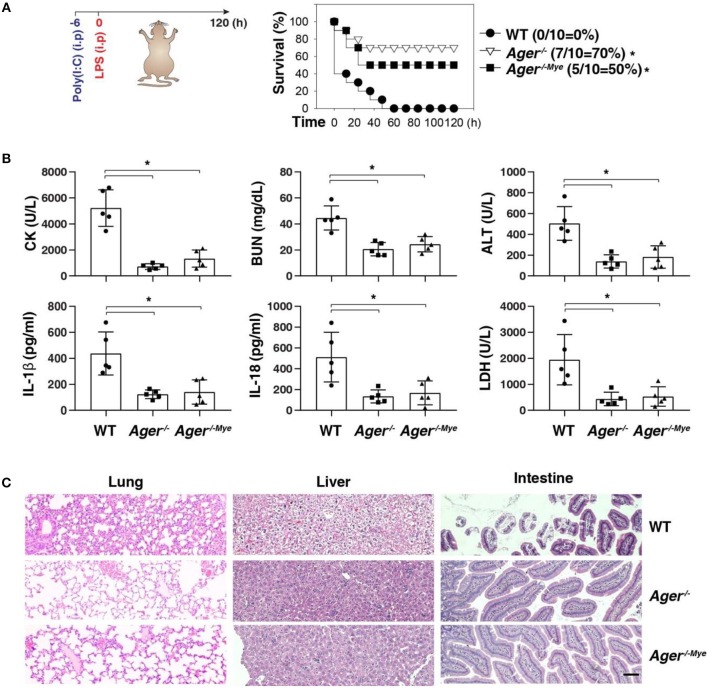
Depletion of AGER protects against septic shock. **(A)** Survival of indicated mice primed with poly(I:C) (10 mg/kg, i.p.) and then challenged 6 h later with LPS (2 mg/kg, i.p.). *n* = 10 mice/group, *P < 0.05, Kaplan-Meier survival analysis. **(B,C)** In parallel to panel A, quantitation of indicated serum markers **(B)** or hematoxylin/eosin staining of indicated tissues **(C)** in poly(I:C)-primed mice challenged with LPS at +3 h (bar = 100 μM). *n* = 5 mice/group, *P < 0.05, ANOVA *LSD* test. Animal data represent two independent experiments.

Next, we investigated whether targeting the AGER-ALOX5 pathway regulates septic shock. We primed mice with poly(I:C) and then re-challenged mice with secondary LPS, which is a classical mouse model of caspase-11 inflammasome-associated septic death (2). Like global knockout of *Ager* (*Ager*^−/−^*)*, the conditional knockout of *Ager* in myeloid cells (*Ager*^−/−*Mye*^*)* also protected mice against secondary LPS-induced death compared to WT mice (Figure 5A). The serum levels of organ dysfunction enzymes (e.g., CK, BUN, and ALT), inflammasome cytokine (e.g., IL-1β and IL-18), and pyroptosis markers (e.g., LDH) were all reduced in *Ager-*deficient mice (Figure 5B). This was also associated with reduced tissue injury (e.g., hemorrhage, leukocyte infiltration, alveolar septal thickening, and edema) in the lung, liver, and intestine (Figure 5C). This genetic *in vivo* evidence indicates that the expression of AGER in myeloid cells play a role in promoting caspase-11-induced endotoxic shock.

**Figure 6:**
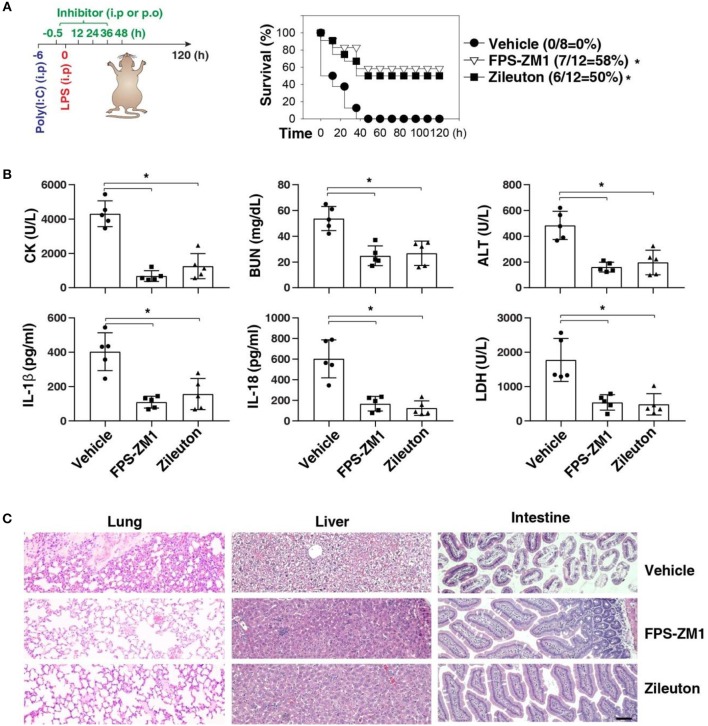
Pretreatment of FPS-ZM1 and zileuton protects against septic shock. **(A)** Survival of indicated mice primed with poly(I:C) (10 mg/kg, i.p.) and then challenged 6 h later with LPS (2 mg/kg, i.p.) in the absence or presence of the administration of FPS-ZM1 (10 mg/kg, i.p.) or zileuton (30 mg/kg, p.o.) at −0.5, +12, +24, +36, and +48 h. *n* = 8–12 mice/group, *P < 0.05, Kaplan-Meier survival analysis. **(B,C)** In parallel to panel A, quantitation of indicated serum markers **(B)** or hematoxylin/eosin staining of indicated tissues **(C)** in poly(I:C)-primed mice challenged with LPS at +3 h (bar = 100 μM). *n* = 5 mice/group, *P < 0.05, ANOVA *LSD* test. Animal data represent two independent experiments.

**Figure 7:**
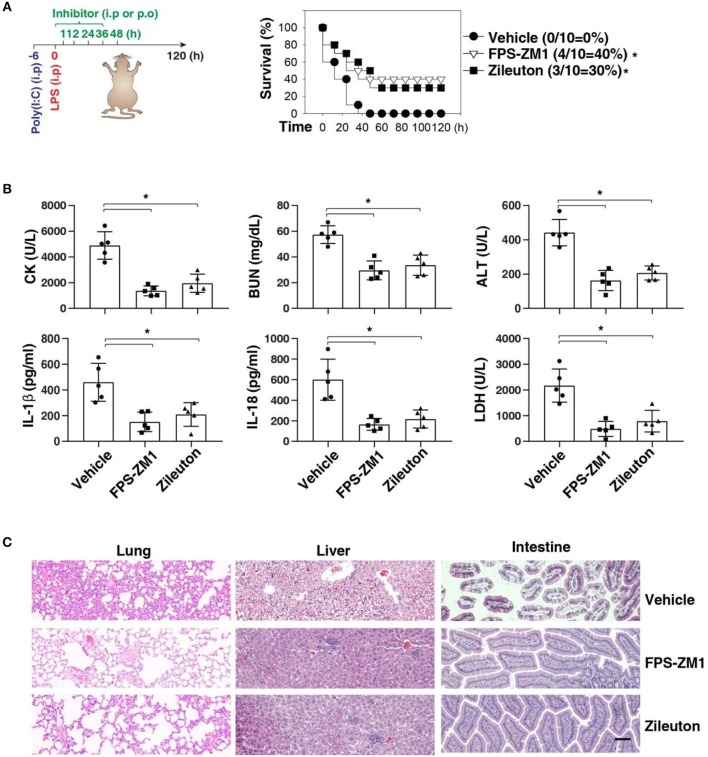
Delayed administration of FPS-ZM1 and zileuton protects against septic shock. **(A)** Survival of indicated mice primed with poly(I:C) (10 mg/kg, i.p.) and then challenged 6 h later with LPS (2 mg/kg, i.p.) in the absence or presence of the administration of FPS-ZM1 (10 mg/kg, i.p.) or zileuton (30 mg/kg, p.o.) at +1, +12, +24, +36, and +48 h. *n* = 10 mice/group, *P < 0.05, Kaplan-Meier survival analysis. **(B,C)** In parallel to panel A, quantitation of indicated serum markers **(B)** or hematoxylin/eosin staining of indicated tissues **(C)** in poly(I:C)-primed mice challenged with LPS at +3 h (bar = 100 μM). *n* = 5 mice/group, *P < 0.05, ANOVA *LSD* test. Animal data represent two independent experiments.

We next sought to evaluate the impact of using AGER inhibitor FPS-ZM1 or ALOX5 inhibitor zileuton in the development of caspase-11-associated sepsis. Indeed, pretreatment with FPS-ZM1 or zileuton significantly protected against LPS lethality in poly(I:C)-primed mice (Figure 6A). The serum levels of CK, BUN, ALT, IL-1β, IL-18, and LDH were all reduced in these mice after the pharmacologic inhibition of AGER or ALOX5 (Figure 6B). As expected, the tissue injury in the lung, liver, and intestine was reduced (Figure 6C). Moreover, delayed administration of FPS-ZM1 or zileuton also increased animal survival (Figure 7A) with decreased serum levels of CK, BUN, ALT, IL-1β, IL-18, and LDH (Figure 7B) as well as the tissue injury in the lung, liver, and intestine (Figure 7C). Taken together, these data indicate that the activation of the AGER-ALOX5 pathway contributes to caspase-11-dependent endotoxic shock.

## Discussion

**Figure 8:**
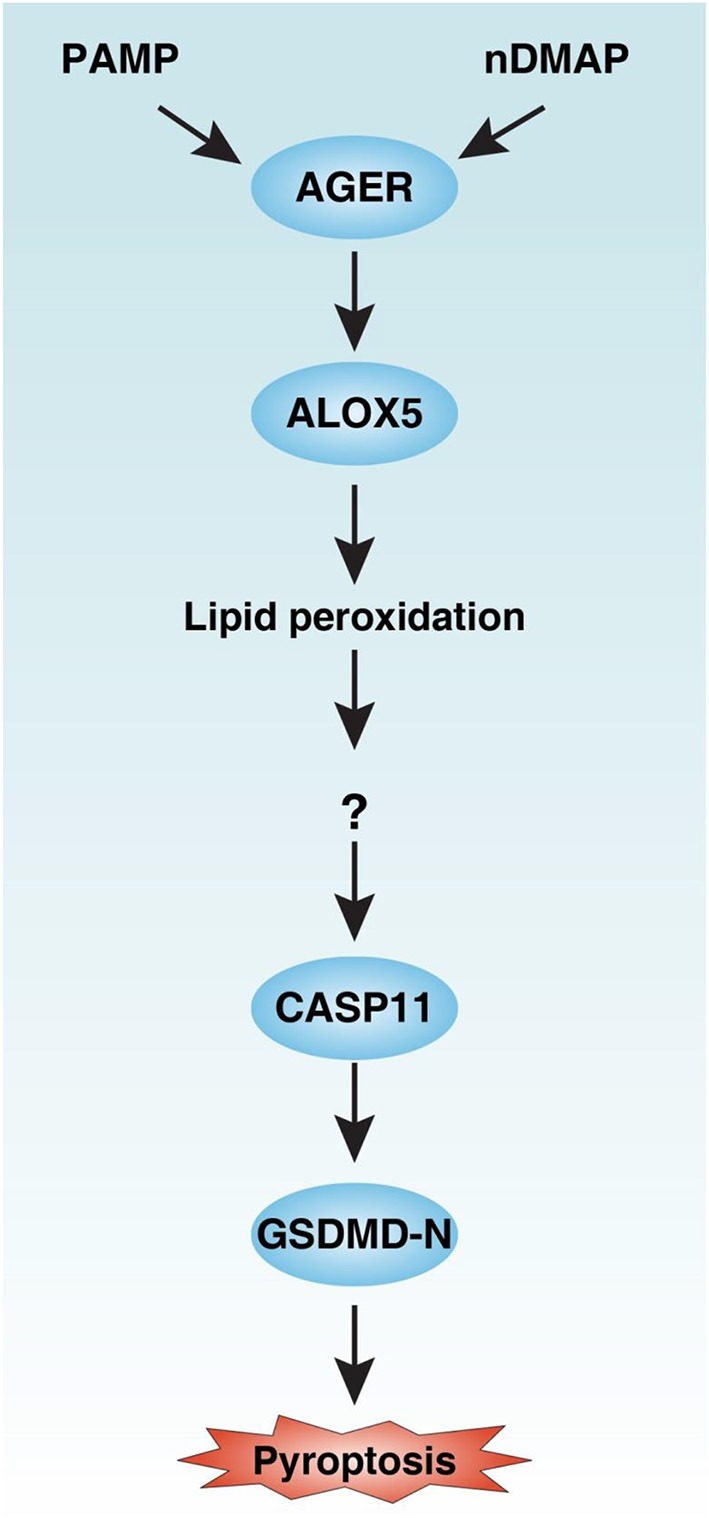
Schematic summary of the role of the AGER-ALOX5 pathway in the regulation of caspase-11 inflammasome activation and pyroptosis. Activation of AGER by PAMP and nDMAP promotes ALOX5-dependent lipid peroxidation. Although the precise mechanism of action of lipid peroxidation remains unknown, the alteration of cellular redox status and the production of lipid peroxides may cause conformational change of CASP11 (27). CASP11 can cleave GSDMD to produce GSDMD-N to mediate pyroptotic cell death. PAMP, pathogen-associated molecular pattern; nDAMP, nuclear damage-associated molecular patterns; AGER, advanced glycosylation end-product specific receptor; ALOX5, arachidonate 5-lipoxygenase; GSDMD-N, N terminal domain of gasdermin D.

Inflammasome, a macromolecular cytosolic protein complex, is a component of the innate immune response to pathogen infection or tissue damage (26). Although inflammasome has been extensively studied over the past decades, the regulation of its underlying significant signaling cascade alterations remains largely unknown. In this study, we demonstrated that AGER-mediated lipid peroxidation is critical for caspase-11 inflammasome activation in macrophages (Figure 8). Consequently, pharmacologic or genetic inhibition of the AGER pathway limits the inflammatory response and improves tissue function and survival in septic mice. Therefore, AGER serves as a checkpoint in caspase-11 inflammasome signals and is a regulator of innate immunity.

Unlike caspase-11 inflammasome, caspase-1-dependent inflammasome is further divided into four major subtypes, namely NLR family pyrin domain containing 1 (NLRP1), NLRP3, NLR family CARD domain containing 4 (NLRC4), and absent in melanoma 2 (AIM2), which can be activated by various PAMPs or DAMPs (20, 28). In contrast, cytosolic LPS has recently been identified as a PAMP that triggers caspase-11 inflammasome activation in macrophages (2–5). Both caspase-1 and caspase-11 can produce the active fragment GSDMD-N at D275 (6, 7). This active fragment then binds to phosphatidylinositol phosphates and phosphatidylserine in the cell membrane inner leaflet to induce pyroptosis in macrophages (9). In contrast, GSDMD-N may be helpful for the clearance of pathogens when they bind to cardiolipin in both the inner and outer leaflets of bacterial membranes (8). Additionally, neutrophil elastase-derived GSDMD-N production at C268 leads to neutrophil death, which may limit the host response to extracellular bacteria (29). These context-dependent findings suggest that various GSDMD-Ns play a dual role in the regulation of the immune response. In the current study, we demonstrated that an nDAMP complex can stimulate caspase-11 inflammasome activation and GSDMD-dependent pyroptosis in macrophages. In addition to passive release after cell death, DAMPs can be actively secreted by immune cells in sepsis (30). Thus, the release of endogenous DAMPs by various cells can amplify the inflammation response and bacterial infection though multiple mechanisms, including the activation of caspase-11 inflammasome in macrophages.

A recent study shows that global knockout of AGER improves survival in mice treated with poly(I:C) followed by LPS, indicating a pathologic role of AGER in the regulation of caspase-11-dependent endotoxemia (31). However, AGER is expressed in multiple immune cells (e.g., T cells and macrophages) and we do not understand which of these cells are critical for the phenotypic attenuation of caspase-11 inflammasome activation in sepsis. Using global depletion or conditional depletion of AGER in myeloid cells in mice, our current results further highlight that AGER in myeloid cells may play a key role in the regulation of caspase-11 inflammasome activation *in vivo*. AGER is a member of the immunoglobulin super family and is predominantly located in the plasma membrane in most cells at baseline (32, 33). Moreover, biologically active AGER can be found in the cytosol and the mitochondrial, nuclear, and extracellular space in response to a variety of stimuli, such as pathogen invasion, oxidative stress, and oncogenic stress (34–36). In addition to nuclear DAMP (HMGB1, histone, and DNA), AGER can directly bind LPS and activate proinflammatory signaling independent of TLR4 (37). We previously demonstrated that AGER contributes to AIM2 inflammasome activation by modulating dsRNA-dependent protein kinase phosphorylation in macrophages during acute pancreatitis (38). Here, we further showed that AGER promotes caspase-11 inflammasome activation by modulating ALOX5-dependent lipid peroxidation in macrophages during sepsis. The functional interplay between AIM2 and caspase-11 inflammasome by AGER remains to be further investigated.

The present study further demonstrates the significance of lipid peroxidation in promoting pyroptosis. Lipid peroxidation contributes to cell death generally through causing serious oxidative damage of cellular membranes. Glutathione peroxidase 4 (GPX4) is an antioxidant enzyme that protects lipid peroxidation. *Gpx4* depletion can induce apoptosis, necroptosis, and ferroptosis as well as pyroptosis, depending on cell type and context (39). In particular, *Gpx4*^−/−^ macrophages are more sensitive to caspase-11 inflammasome activation in response to cytosolic LPS signaling (40). Accordingly, the conditional knockout of *Gpx4* (*Gpx4*^−/−*Mye*^*)* in myeloid cells increases the risk of polymicrobial sepsis through the activation of caspase-11 inflammasome (40). In contrast, ALOX5 is an enzyme in the metabolism of arachidonic acid into leukotrienes, the lipid mediators involved in inflammation, aging, and several allergic conditions (41). *Alox5*^−/−^ mice exhibit suppressed inflammation in response to infection or tissue injury (42, 43). We show here that AGER-mediated ALOX5 activation promotes lipid peroxidation, IL-1β release, and pyroptosis in macrophages in response to DAMPs and PAMPs, supporting a model in which ALOX5 is a key lipoxygenase in the control of caspase-11 inflammasome.

Septic shock remains a medical challenge with poor clinical outcomes. Sepsis subtypes can be identified based on different inflammasome activation patterns (44). Current studies summarize preclinical evidence suggesting that FPS-ZM1 or zileuton might be a useful target for the treatment of sepsis, especially caspase-11-associated sepsis. FPS-ZM1 is a blood-brain barrier permeant, non-toxic, and high affinity AGER-specific inhibitor used in experimental chronic obstructive pulmonary disease (COPD) and Alzheimer's disease (24, 45). Zileuton is an inhibitor of ALOX5 and is used to prevent asthma attacks in adults and children (25, 46). Direct evidence that the caspase-11 inflammasome is indeed driving COPD, Alzheimer's disease, and asthma remains to be clearly established.

In summary, our results indicate that AGER plays a novel role in caspase-11 inflammasome activation, in part by regulating ALOX5-dependent lipid peroxidation in macrophages. This axis contributes to systemic inflammation and tissue injury after the onset of sepsis. A previous study showed that the global knockout of AGER protects against sepsis (47). Our study further demonstrates that conditional depletion of AGER in myeloid cells prevents septic death, supporting that AGER expression in myeloid cells, including macrophages, is important for the innate immune response (48–50).

## Ethics Statement

Animal studies were approved by our Institutional Animal Care and Use Committees and conducted in accordance with Association for Assessment and Accreditation of Laboratory Animal Care guidelines (http://www.aaalac.org/).

## Author Contributions

DT, BZ, and RK designed the experiments. RC, LZ, SZ, BZ, RK, and DT conducted the experiments. DT and RK wrote the paper. QW and YS provided important reagents.

### Conflict of Interest Statement

The authors declare that the research was conducted in the absence of any commercial or financial relationships that could be construed as a potential conflict of interest.
